# Birth Size and Maternal, Social, and Environmental Factors in the Province of Jujuy, Argentina

**DOI:** 10.3390/ijerph19020621

**Published:** 2022-01-06

**Authors:** Jorge Ivan Martinez, Marcelo Isidro Figueroa, José Miguel Martínez-Carrión, Emma Laura Alfaro-Gomez, José Edgardo Dipierri

**Affiliations:** 1 Instituto de Ecorregiones Andinas (INECOA) CONICET-UNJu, San Salvador de Jujuy 4600, Argentina; fmi_753@hotmail.com (M.I.F.); ealfaro@inbial.unju.edu.ar (E.L.A.-G.); 2 Departamento de Genética y Bioantropología, Instituto de Biología de la Altura (InBiAl), National University of Jujuy, San Salvador de Jujuy 4600, Argentina; jedjujuy@gmail.com; 3 Departamento de Biología Molecular, Instituto de Estudios Celulares, Genéticos y Moleculares (ICeGeM), National University of Jujuy, San Salvador de Jujuy 4600, Argentina; 4 Department of Applied Economics, Faculty of Economics and Business, Murcia University, 30100 Murcia, Spain; jcarrion@um.es

**Keywords:** birth size, maternal factors, altitude, Jujuy, Argentina

## Abstract

Introduction: birth size is affected by diverse maternal, environmental, social, and economic factors. Aim: analyze the relationships between birth size—shown by the indicators small for gestational age (SGA) and large for gestational age (LGA)—and maternal, social, and environmental factors in the Argentine province of Jujuy, located in the Andean foothills. Methods: data was obtained from 49,185 mother-newborn pairs recorded in the Jujuy Perinatal Information System (SIP) between 2009 and 2014, including the following: newborn and maternal weight, length/height, and body mass index (BMI); gestational age and maternal age; mother’s educational level, nutritional status, marital status and birth interval; planned pregnancy; geographic-linguistic origin of surnames; altitudinal place of birth; and unsatisfied basic needs (UBN). The dataset was split into two groups, SGA and LGA, and compared with adequate for gestational age (AGA). Bivariate analysis (ANOVA) and general lineal modeling (GLM) with multinomial distribution were employed. Results: for SGA newborns, risk factors were altitude (1.43 [1.12–1.82]), preterm birth (5.33 [4.17–6.82]), older maternal age (1.59 [1.24–2.05]), and primiparous mothers (1.88 [1.06–3.34]). For LGA newborns, the risk factors were female sex (2.72 [5.51–2.95]), overweight (1.33 [1.22–2.46]) and obesity (1.85 [1.66–2.07]). Conclusions: the distribution of birth size and the factors related to its variability in Jujuy are found to be strongly conditioned by provincial terrain and the clinal variation due to its Andean location.

## 1. Introduction

Birth size is affected by diverse maternal, environmental, social, and economic factors, including maternal health problems that can develop during pregnancy. Among the maternal factors, ethnicity, age, parity, and education level stand out [1,2,3]. Among the environmental factors, higher altitude is one of the most obvious. Globally, the likelihood of adverse perinatal outcomes—including low birth weight (<2500 g) and small for gestational age (SGA)—increases in high-altitude pregnancies [4]. Interactions among these maternal and non-maternal factors with fetal growth are explained by the theory of maternal capital, defined by Wells [5] as any maternal characteristic (biological, cultural, economic, social) that can be invested in the fetus and whose accumulation enables the mother to protect the fetus from adverse environmental conditions.

Birth weight is the universal indicator for evaluating newborn (NB) size, although SGA, appropriate for gestational age (AGA), large for gestational age (LGA), weight-length ratio, body mass index (BMI), and ponderal index at birth are also used. There is no consensus in neonatal anthropometry on available growth charts because the charts differ in their criteria for inclusion and exclusion, measuring techniques and instruments, precision in the evaluation of gestational age, and methods for calculating percentiles [6]. The publication of INTERGROWTH-21st standards [7] partially resolves these issues.

One of the best indicators for capturing the Gaussian distribution of birth size with birth weight at vertex, “optimal birth weight”, is the classification of newborn infants based upon gestational age and birth weight proposed by Battaglia and Lubchenco [8], which distinguishes three categories of size at birth: (1) SGA newborns are those smaller than normal for their gestational age, most commonly defined as a weight below the 10th percentile for their gestational age; (2) LGA newborns are those larger than normal for their gestational age, defined as a weight greater than the 90th percentile for their gestational age; and (3) AGA newborns are those with birth weights between the 10th and 90th percentiles.

The province of Jujuy, in northwestern Argentina, is located on the eastern flank of the Andes and spans foothills ranging in altitude from about 500 m to over 4000 m. This altitudinal cline has conditioned the placement of human settlements within four well-defined ecological regions: (1) Puna (>3500 m), (2) Quebrada (about 2500 m), (3) Valleys (about 1500 m), and (4) Ramal (<500 m). Jujuy populations—particularly those located in highlands (>2500 m)—show a cultural and ethnic affinity with other peoples of the Andean world, which is manifested in onomastics and genetic traits. A significant percentage of individuals (close to 20% carry indigenous surnames (from Quechua, Aymara, and now-extinct original Andean languages) [9,10]. On the other hand, molecular studies have shown that the least mixed Jujuy populations, dominated by a strong indigenous genetic component, are located in the Puna and Quebrada regions. This has been demonstrated using different uni- or biparental markers [11,12,13] and Next Generation Sequencing [14].

Previous studies of Jujuy populations indicate an association between high altitude and lower birth weight. Compared to infants born at lower altitudes, highland infants are more likely to present stunting, reduced height and body surface area, and ectomorphy, as well as increased BMI, ponderal index scores, and body surface area/mass ratios [15,16,17,18]. More recently, Martínez et al. [3] have shown that infants born above 2500 m have birth weights significantly lower than infants born below that altitude and that, of the array of maternal characteristics they examined (age, anthropometry, nutritional status, obstetric history, education, marital status, pregnancy planning, and unsatisfied basic needs), only maternal anthropometric variables protected newborns against the negative impact of highland environments. In the same study, the prevalence of SGA newborns was significantly lower in Puna and Quebrada regions, the opposite of what was observed for LGA newborns [3].

This study’s goal was to analyze the maternal, social, and environmental factors that affected birth size of SGA and LGA newborns in Jujuy between 2009 and 2014.

## 2. Methods and Materials

This was an observational, analytical, and retrospective study of consecutive births registered by the Perinatal Informatics System (SIP) of Jujuy’s Ministerio de Salud. SIP is a tool developed by the Centro Latinoamericano de Perinatología, Salud de la Mujer y Reproductiva for collecting data on mothers and newborns in neonatal and obstetric centers. SIP is used in all of the provincial public health system’s maternity wards. In this paper, data collected between 2009 and 2014 was analyzed. The original SIP database (without applying exclusión criteria) covers 70% of births in Jujuy province (Supplementary Material—Figure S1).

This study followed the bioethical guidelines issued by Argentina’s health ministry (Ministerio de Salud, 2011 [19]), which state that epidemiological studies analyzing public-records data or data already available to the public do not require further revision or approval by an ethics committee. Nonetheless, the newborn and mother information was anonymized prior to analysis.

### 2.1. Data and Variables

The following exclusion criteria was used: (1) gestational age < 24 + 0 weeks and > 42 + 6 weeks; (2) simultaneous lack of data on weight, height, gestational age, sex, and maternal place of residence during pregnancy; (3) twin pregnancy; (4) congenital malformation; and (5) birth weight and height > P97 of INTERGROWTH-21st standard for gestational weight and height (Villar et al., 2014 [7]). Alexander’s criterion was applied to correct incompatibilities between birth weight and gestational age [20].

Fetal variables were the indicators for SGA and LGA, calculated using the 10th and 90th percentiles of the INTERGROWTH-21st standard (Villar et al., 2014 [7]). For newborns, weight (kg), length (cm), and gestational age were analyzed; while in mothers, weight (kg), height (cm), pre-conception BMI (kg/m^2^), age, and percentage of homes with unsatisfied basic needs (UBN) in the mother’s place of residence were analyzed.

The prevalence of SGA, AGA, and LGA newborns was analyzed in relation to various categories. Altitude of the maternal place of residence was divided between highlands (HL) > 2500 m, and lowlands (LL) < 2500 m. Regarding parity, two categories were established: primiparous and multiparous. Three groups of birth intervals were defined: <18 months, 19 to 60 months, and >60 months. The pregnancy planning variable was divided into two groups, with and without planning. Pregnant women were grouped according to their levels of education: without schooling, primary, secondary, and university. The following categories of maternal marital status were defined: married, stable union, single, and other. Given that surname-based ethnicity classification systems have proven highly efficient and congruent within northwest Argentine populations, ethnicity was established from the two maternal surnames, which were classified, according to their geographic-linguistic origins, in two large categories: native, that is to say, of indigenous or American origin, and foreign, arising in other parts of the world [9,21]. Using BMI and World Health Organization (WHO) criteria, the following categories of maternal nutritional status were defined: underweight (BMI ≤ 18.5), normal (18.5 ≤ BMI ≤ 25), overweight (25 ≤ BMI ≤ 30), and obese (BMI ≥ 30). Finally, whether or not the newborn was preterm was established.

With regards to the variable for the home or locality of the pregnant woman, as recorded by SIP, covariables of altitude and percentage of the population with UBN were established. The latter, an indicator of critical shortcomings and populational poverty, uses information directly related to four areas of basic human needs (housing, health service, basic education, and minimum income). Data for UBN came from the 2010 national census (Instituto Nacional de Estadísticas y Censos).

### 2.2. Statistical Analysis

Category variables were expressed as percentages, and statistics differences were calculated with a Chi-squared test using SPSS software (V.22, IBM Company, Armonk, NY, USA). For continuous fetal and maternal variables, means ± standard deviation (SD) were calculated. Comparisons of these variables for SGA and LGA infants were assessed using ANOVA.

A spatial autocorrelation analysis of the incidences of SGA and LGA newborns—according to maternal municipal residences, using Moran’s Index—was carried out. Spatial association patterns were estimated by calculating local indicators of spatial association (LISA, Anselin, 1995 [22]) and local spatial clusters from hot spots and cold spots in groupings of high and low values. The high-high clusters (red) and the low-low clusters (dark blue) indicate the presence of local positive spatial autocorrelations; while the high-low clusters (pink) and low-high clusters (light blue) are atypical spatial values that indicate local negative spatial autocorrelations.

To integrate the relationship between the presence/absence of SGA or LGA infants as a function of newborn, maternal, and socioeconomic variables, general lineal modeling (GML) with a multinomial distribution was used, with AGA infants as the reference category. By means of regression selection, a minimum adequate model was attained, taking into account variations in the AIC (Akaike information criteria) values of the model when each variable that makes up the fixed effects was individually removed. The variables originally used were the following: sex, gestational age, altitude, surname, maternal age, parity, birth interval, planned pregnancy, educational level, marital status, unsatisfied basic needs, and BMI. Odds ratios (ORs) and confidence intervals of 95% are reported. R software (R Core Team, 2019 [23]), the RStudio interface (RStudio Team, 2015 [24]), and the “nnet” statistic package (Venables and Ripley, 2002 [25]) were used for the analysis.

## 3. Results

After applying exclusion criteria in percentage terms of the SIP registers chosen for this study, they represent 60% of live births in Jujuy province, 49,185 mother-child pairs—25,014 boys, 24,180 girls (Supplementary material—Figure S1). Table 1 shows the number of individuals and prevalence of SGA, LGA, and AGA newborns by highland and lowland regions. A greater prevalence of SGA newborns in highland regions is observed; the opposite for LGA newborns.

The study found 1965 SGA newborns; 38,192 AGA newborns; and 9028 LGA newborns. Their distributions are presented in Table 2, Table 3 and Table 4. SGA newborns and their mothers had significantly lower average weight, length/height, and BMI than the AGA groups (Table 2). Mean maternal age did not show statistically significant differences between SGA and AGA newborns, but gestational age did. Gestational age varied significantly among SGA, AGA, and LGA newborns, but with the greatest variation among AGA newborns (Table 2).

Greater prevalence of SGA newborns was observed in mothers with at least one of the following characteristics: highland residence, primiparity, long birth-interval (>60 months), optimal pre-conception nutritional status, and preterm birth, with statistically significant differences only for the last three variables. AGA newborns were, in comparison with SGA newborns, more prevalent among the remaining variables, but the differences were only significant in lowland mothers, multiparous mothers, and mothers that did not have preterm births (Table 3).

The prevalence of LGA newborns, in comparison with AGA newborns, was significantly greater in mothers with at least one of the following characteristics: lowland residence, multiparity, planned pregnancy, married, stable partner, foreign surname, overweight, and obese. In contrast, significantly higher prevalence of AGA newborns was found in mothers with at least one of the following characteristics: highland residence, primiparity, unplanned pregnancy, single, and indigenous surnames (Table 3). Maternal education was the only variable that did not significantly influence any of the birth-size categories.

**Figure 1:**
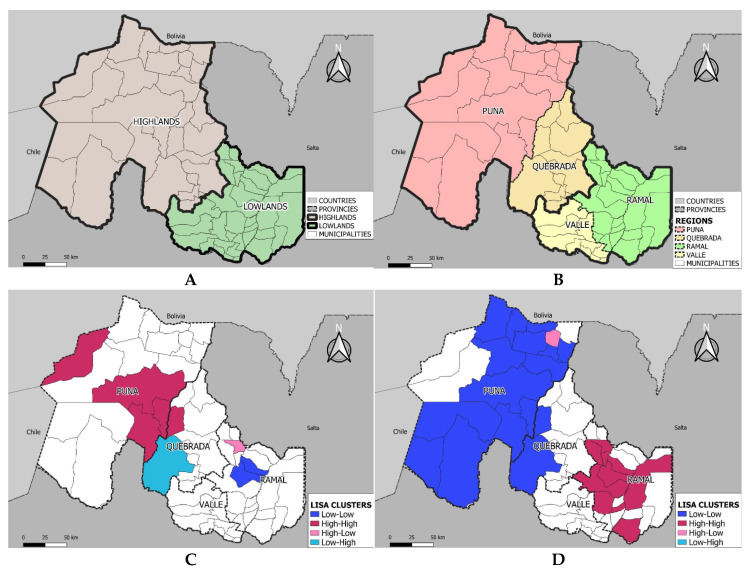
Distribution by Altitude, Region, and Cluster Groupings of Newborn Birth Size in the Province of Jujuy. (**A**) Altitudinal regions of the province of Jujuy; (**B**) Ecoregions of the province of Jujuy; (**C**) Clusters of small for gestational age in the province of Jujuy; (**D**) Clusters of large for gestational age in the province of Jujuy.

Figure 1A shows the breakdown by altitude (highland and lowland) in Jujuy, while Figure 1B shows the breakdown by municipalities of maternal residence. The raw Moran’s Index was 0.111 for SGA newborns and 0.699 for LGA newborns. Figure 1C shows prevalence clusters for SGA newborns, using the LISA method; Figure 1D shows prevalence clusters for LGA newborns, also using the LISA method.

Figure 1C provides evidence of two clusters with positive spatial autocorrelation. The larger cluster corresponds to greater prevalence of SGA newborns and is located in highland municipalities, particularly in the Puna region. The smaller cluster includes two municipalities with significantly lower prevalence of SGA newborns, which are located in the lowlands. Figure 1D shows the opposite pattern, with larger clusters and positive spatial autocorrelations for LGA newborns. Lower prevalence of LGA newborns is seen in the dark blue cluster of the Puna (highlands), while greater prevalence of LGA newborns is located in the Ramal region (lowlands).

According to GLM with multinomial distribution and Akaike’s criteria, newborns had a significantly greater risk of being born SGA when they were born preterm and when their mothers were older, highland residents, or primiparous. The only protective factor for SGA newborn was the female sex. On the other hand, the risk of being born LGA was double in the female sex, and it was confirmed that increases in maternal BMI also raised the chance of being born with this phenotype. The factors that acted as protection against LGA were that the mother resided in the highlands and the low preconception weight. (Table 4).

## 4. Discussion

Birth size is an important outcome of a healthy pregnancy that reflects critical aspects of fetal and maternal health, is strongly associated with perinatal mortality and morbidity, and influences long-term health of the individual [26]. The distribution of birth size has two critical zones, the left and right tails, which express the maximum pressure of normalizing or stabilizing selection.

This study verifies that in Jujuy the distribution of birth size and the factors related to its variability are strongly conditioned by provincial orography and the altitudinal cline that are characteristic of the Andean foothills. Based on these considerations, it can be argued that the spatial distribution of SGA newborns in Jujuy is generally a mirror image of the distribution of LGA newborns, and therefore that the explanations and interpretations of both phenotypes are, at the same time, complementary and opposite.

The interpretation of the three indicators of birth size should be undertaken while considering variations in birth weight according to Jujuy’s geographic regions evidenced in these studies [16,18,27]. These studies show asymmetry and highly significant leptokurtosis in the distributions of birth weight in the four geographic regions, where leptokurtosis increases proportionally with altitude. A population’s distribution of birth weight can shift to higher or lower weights, and the optimal birth weight can be lower in populations with a lower average birth weight. Beall [28], while analyzing optimal birth weights in Peruvian populations at different altitudes, found that at 3860 m the difference between optimal birth weight and average birth weight was 322 g, while, for a similar population at 600 m, the difference was 222 g. This suggests that populations at lower altitudes are closer to their optimal birth weights than those at higher altitudes. The greater proportions of SGA and AGA newborns—and the lower proportions of LGA newborns—in highland regions (Puna and Quebrada), and their spatial representation, as shown in Figure 1C and D, are likely a reflection of adaptive mechanisms to high-altitude ecosystems and biological traits selectively acquired over time. It is worth mentioning that these adaptive processes are not recognized by international standards which, as exclusion criteria, use mothers’ residency at an altitude > 1500 m and mothers’ height < 155 cm [7,29]. From these observations, the arbitrariness of the 10th and 90th percentile cut-off points for birth weights, normally used to define SGA and LGA newborns, is questioned, while the creation of local growth charts, adjusted for various confounding factors, to define birth size, is encouraged [1]. This situation becomes more critical in high-altitude environments where the effects of hypoxia on pre- and post-natal growth constitute an omnipresent negative factor that cannot be controlled by any cultural adaptation [30].

In summary, this study confirms previous findings which show that increased altitude lowers birth weight and raises the incidence of SGA newborns [3,31]. Although high altitude constitutes a risk factor for SGA, the prevalence of this indicator in Jujuy highlands is lower than the prevalence observed across Argentina in 2012 (7.6; 6.3 ± 16.6) [32] and in 2013 (9.9) [33]. With regards to the LGA indicator, there are no national estimates or global estimates [34] that include all countries, which would allow comparisons with this study’s results. However, we can state that this phenotype is more frequent in lowland areas, particularly in the Ramal region (Table 1, Figure 1D).

Maternal height, pregestational maternal weight, and weight increase during pregnancy have been identified as the main maternal capital components [5] which determine fetal growth patterns and, consequently, the size of newborns [35]. Previous studies in Jujuy have shown that maternal weight, height, and BMI were positively correlated to equivalent newborn variables, and that all these anthropometric variables were inversely correlated to altitude [36]. The close relationship between maternal anthropometry and fetal size, evaluated with SGA and LGA newborns, is made evident by the variation in prevalence of these indicators, showing that there is a clinically significant difference in birth weight percentiles when they are stratified by maternal height and weight [37].

The kurtosis and symmetry in the distribution of birth weight as a function of altitude could also explain differences by sex in the prevalence of SGA and LGA newborns in bivariate analysis. Independently of any biological or environmental factor, average birth weight is significantly greater for the male sex than for the female sex. Therefore, the distribution of birth weights for the female sex is expected to be more skewed to the left in the highlands and that P10 values will be lower. The opposite would be expected to occur for the male sex in the lowlands, thus explaining the greater incidence of SGA newborns in the female sex and of LGA newborns in the male sex that were observed in bivariate analysis. Nonetheless, GLM shows that the female sex has a significantly greater odds ratio for LGA. There is no reasonable explanation, nor precedents in the literature, for this finding.

Compared with AGA newborns, the mean gestational age for SGA newborns was significantly lower (Table 2). This translates into a greater prevalence of preterm births in this group (5.4% versus 18.1%, respectively; Table 3) and a result for GLM indicating that preterm birth constitutes a risk factor for SGA (Table 3). In Latin America and the Caribbean region in 2010, the prevalence of preterm-birth SGA newborns was 1.8% (1.4–2.5%) [38].

Bivariate analysis indicates that, in relation to AGA, the prevalence of SGA—excluding altitude, sex, and preterm birth—is likely associated with a few relevant maternal and obstetric conditions, or ones that are commonly attributed as risk factors for SGA (maternal malnutrition, marital status, and educational level), highlighting multiparity, longer birth intervals, and optimal maternal nutritional status. According to Kozuki et al. [39], even if nulliparous, minor (<18 years old) mothers have a greater probability of SGA newborns, this risk will also be seen in older, multiparous mothers, as shown in bivariate analysis. The birth of a first child leads to important physiological, social, and wellbeing changes, which require adaptive mechanisms based on social support. Such support could be weakened for later births, especially for mothers of low socioeconomic or educational levels [37]. However, after GLM, primiparity emerges as the only risk factor for SGA.

The relationship between longer birth intervals and risk of SGA is debatable. While Cass et al. [40] contend that an unusually long interpregnancy interval is associated with a moderate independent effect of elevated odds of SGA, Kozuki et al. [39] conclude that birth intervals shorter than 18 months are significantly associated with SGA, preterm birth, and death in the first year of life. It is hypothesized that the mother’s anatomical and physiological capability to handle the growth of the fetus may revert to a state of nulliparity if there has been a long period since her last pregnancy, and thus she may present the outcomes of a nulliparous mother [39].

GLM indicates that older mothers constitute a risk factor for SGA newborns. Pregnancy at later ages is a condition that has grown in recent years. In the United States, during the decade 1991–2001, the number of pregnancies among women aged 35 to 39 rose 36%, and among women aged 40 to 44, pregnancies rose 70% [41]. In developed countries, the delay of maternity is related to women’s new perspectives on work and professional life and to reproductive problems, such as infertility. A recent study of a retrospective cohort of 2009–2013 births (*n* = 17,031,005) by the National Vital Statistics System in the United States apparently shows that the frequency of SGA newborns was significantly higher in nulliparous women ≥ 30 years old and in all women ≥ 40 years old, when compared to women aged 20 to 29 [42]. The authors argue that the association between SGA and mother’s age is a positive dose-response relationship, and, although the exact mechanism of the association has not been demonstrated, the underlying factor could be a deficient exchange of oxygen.

Regarding the bivariate analysis of LGA with respect to AGA, significant differences associated with maternal nutritional status, marital status, parity, and ethnicity are found. Excessive maternal adiposity (obesity and overweight) constitute a risk factor for LGA, while deficient adiposity (underweight) exerts a protective effect. According to Chiavaroli et al. [43], greater neonatal adiposity—and thus greater birth size—represents the expression of a complex fetal-maternal interaction, driven by fetal genetic factors and by the intrauterine environment, which manifests as a nutritional excess within the uterus that very likely reflects maternal nutrition, particularly obesity. As for marital status and its relationship to LGA, married mothers and mothers with stable unions probably have better food opportunities and emotional support than single mothers, with lower prevalence of LGA newborns compared to AGA newborns.

Differences in the prevalence of SGA and LGA newborns according to type of surname (indigenous or foreign) in Jujuy populations are probably related to ethnic variations, altitude, and maternal BMI. Jujuy populations with indigenous surnames, particularly populations in the highlands, have lower average weights and heights, lower BMIs, and are less mixed ethnically than populations in the lowlands [10,11,12,13,14,15,36]. Risk factors found in the bivariate analysis between AGA and LGA are probably related to maternal BMI, the only risk factor for LGA that GLM tion returns.

One of the strengths of this study is its contribution to public health by increasing knowledge about the diverse factors—and their prevention—that affect birth size when evaluated with indicators that relate birth weight with gestational age in high-altitude environments, such as in the province of Jujuy. Both SGA and LGA are related to an increase in infant mortality. Non-macrosomic LGA newborns (birth weight < 4000 g) are likely to show an increase in mortality and morbidity, even with non-diabetic mothers. On the other hand, in low- and middle-income countries, about one in five infants are SGA, and one in four neonatal deaths are among such infants [38].

The study’s main limitation is that the data come only from the public health system and does not include deliveries that occurred in privately managed institutions. Information on maternal health during pregnancy, such as the occurrence of eclampsia or diabetes, could not be analyzed due to the lack of availability of appropriate data. These health conditions have been observed to affect birth size. This is particularly true for mothers located in the most remote areas of the province of Jujuy, which makes laboratory access and consultation with specialists impossible. Using surnames as an indicator of ethnicity may lead to misclassification. However, studies on the association of surnames with genetic markers reveals that there is a reasonable agreement of ethnic classification of individuals by surname and phenotype data on genetic markers [44,45].

## 5. Conclusions

The relationship between birth size—evaluated by the indicators for SGA and LGA—and maternal, social, and environmental factors in the province of Jujuy show that these factors differ according to the indicator. For SGA, the risk factors are altitude, preterm birth, later maternal age, and primiparity, while for LGA, the risk factors are female sex and higher BMI.

## Figures and Tables

**Table 1:** Proportional distribution of Newborns by Birth Size, Altitude, and Geographic Region.

Variable	Category	SGA			AGA			LGA		
		*n*	%	95% CI	*n*	%	95% CI	*n*	%	95% CI
Altitude	Lowland	1631	3.8	3.6–4	32,503	76.2	75.8–76.6	8509	20	19.6–20.3
	Highland	334	5.1	4.6–5.7	5689	87	86.1–87.8	519	7.9	7.3–8.6
Region	Puna	216	4.8	4.2–5.4	3999	88.1	87.2–89	323	7.1	6.4–7.9
	Quebrada	166	5.5	4.7–6.3	2562	84.3	83–85.6	311	10.2	9.2–11.3
	Valle	1108	3.9	3.7–4.1	21,774	76.4	75.9–76.9	5609	19.7	19.2–20.2
	Ramal	475	3.6	3.3–4	9857	75.1	74.4–75.9	2785	21.2	20.5–21.9

Footnote: Small for gestational age (SGA), Appropriate for gestational age (AGA), Large for gestational age (LGA).

**Table 2:** Mean and Standard deviation (SD) of Maternal and Newborn Variables by Birth-Size Categories.

Variable	SGA (*n* = 1965)		AGA (*n* = 38,192)		LGA (*n* = 9028)		*p-*Value
	$\bar{x}$	SD	$\bar{x}$	SD	$\bar{x}$	SD	
Newborn Weight	2.37	0.55	3.23	0.46	3.80	0.47	0.000
Newborn Length	46.97	3.92	50.05	2.75	51.39	2.60	0.000
Gestational Age	38.78	2.79	39.17	1.69	38.68	1.62	0.000
Maternal Weight	55.59	11.22	57.36	11.24	61.76	12.54	0.000
Maternal Height	1.54	0.06	1.55	0.06	1.56	0.06	0.000
Maternal Body Mass Index	23.36	4.56	23.89	4.52	25.27	5.03	0.000
Maternal Age	24.57	6.92	24.76	6.42	26.18	6.43	0.222
Unsatisfied Basic Needs (%UBN)	17.16	6.29	17.60	6.95	17.37	7.04	0.008

**Table 3:** Proportional distribution of SGA, AGA and LGA Newborns by Environmental, Maternal, and Newborn Variables.

Variable	Category	SGA		AGA		LGA		*p-*Value AGA vs. SGA	*p-*Value AGA vs. LGA
		*n*	%	*n*	%	*n*	%		
Altitude	Lowland	1632	82.9	32,502	85.1	8509	94.3	0.0152	0.0001
	Highland	336	17.1	5683	14.9	519	5.7	0.2728	0.0001
	Total	1968	100	38,185	100	9028	100	-	
Parity	Multiparous	931	71	22,351	74.8	6342	83.5	0.0090	0.0001
	Primiparous	381	29	7546	25.2	1250	16.5	0.0964	0.0001
	Total	1312	100	29,897	100	7592	100	-	
Birth Interval	<18 months	138	16.3	4148	20.8	1132	20.1	0.1990	0.6061
	19–60 months	6	0.7	149	0.7	46	0.8	1.000	0.9444
	>60 months	704	83	15,607	78.4	4456	79.1	0.0036	0.3153
	Total	848	100	19,904	100	5634	100	-	
Planned Pregnancy	No	1046	64.6	19,418	62.5	4372	60.3	0.1715	0.0068
	Yes	572	35.4	11,630	37.5	2877	39.7	0.3109	0.0295
	Total	1618	100	31,048	100	7249	100	-	
Educational Level	Elementary	508	26.9	9939	27.2	2284	26.6	0.8822	0.5607
	Secondary	1225	64.9	23,738	65.0	5623	65.4	0.9430	0.5716
	University	137	7.3	2593	7.1	631	7.3	0.9293	0.8611
	No Schooling	17	0.9	273	0.7	62	0.7	0.9243	10,000
	Total	1887	100	36,543	100	8600	100	-	
Marital Status	Married	124	6.9	2821	8.1	845	10.3	0.6308	0.0455
	Stable Union	1048	58.1	21,058	60.2	5358	65.1	0.1754	0.0001
	Single	629	34.8	11,040	31.5	2014	24.5	0.0836	0.0001
	Other	4	0.2	88	0.3	17	0.2	0.9714	0.9437
	Total	1805	100	35,007	100	8234	100	-	
Surname	Foreign	1550	78.8	29,532	77.3	7206	79.8	0.1689	0.0001
	Indigenous	418	21.2	8649	22.6	1821	20.2	0.5035	0.0251
	Total	1968	100	38,181	100	9027	100	-	
Maternal Pre-Conception Anthropometry	Underweight	87	5.8	1327	4.4	202	2.7	0.5413	0.2606
	Optimal	1002	67.1	19,351	63.7	3911	53.2	0.0289	0.0001
	Overweight	272	18.2	6832	22.5	2095	28.5	0.0950	0.0001
	Obesity	133	8.9	2880	9.5	1146	15.6	0.8173	0.0001
	Total	1494	100	30,390	100	7354	100	-	
Preterm	Yes	357	18.1	2052	5.4	516	5.7	0.0001	0.7887
	No	1611	81.9	36,137	94.6	8512	94.3	0.0001	0.2730
	Total	1968	100	38,189	100	9028	100	-	

**Table 4:** General Lineal Model (GML) Results for SGA and LGA Newborns.

Fixed Effects		OR (95% CI)	
		SGA	LGA
Sex	Male	1	1
	Female	0.47 (0.38–0.57)	2.72 (2.51–2.95)
Preterm	No	1	1
	Yes	5.33 (4.17–6.82)	1.06 (0.88–1.28)
Altitude	Lowland	1	1
	Highland	1.43 (1.12–1.82)	0.35 (0.3–0.41)
Maternal Age	Optimal	1	1
	Adolescent	0.7 (0.47–1.05)	0.87 (0.74–1,03)
	Older	1.59 (1.24–2.05)	1.09 (0.96–1.23)
Parity	Multiparous	1	1
	Primiparous	1.88 (1.06–3.34)	1.21 (0.88–1.67)
BMI	Underweight	1.11 (0.7–1.74)	0.66 (0.52–0.84)
	Optimal	1	1
	Overweight	0.92 (0.74–1.15)	1.33 (1.22–2.46)
	Obesity	1.16 (0.88–1.53)	1.85 (1.66–2.07)

## Data Availability

Data sharing not applicable.

## Supplementary Materials

The following supporting information can be downloaded at: https://www.mdpi.com/article/10.3390/ijerph19020621/s1, Figure S1: Database construction.
