# Supplementary figures and images for: Birth Size and Maternal, Social, and Environmental Factors in the Province of Jujuy, Argentina
Source: Int J Environ Res Public Health. 2022 Jan 6;19(2):621. doi: 10.3390/ijerph19020621 (PMC8775369; doi:10.3390/ijerph19020621)

## Supplementary Material

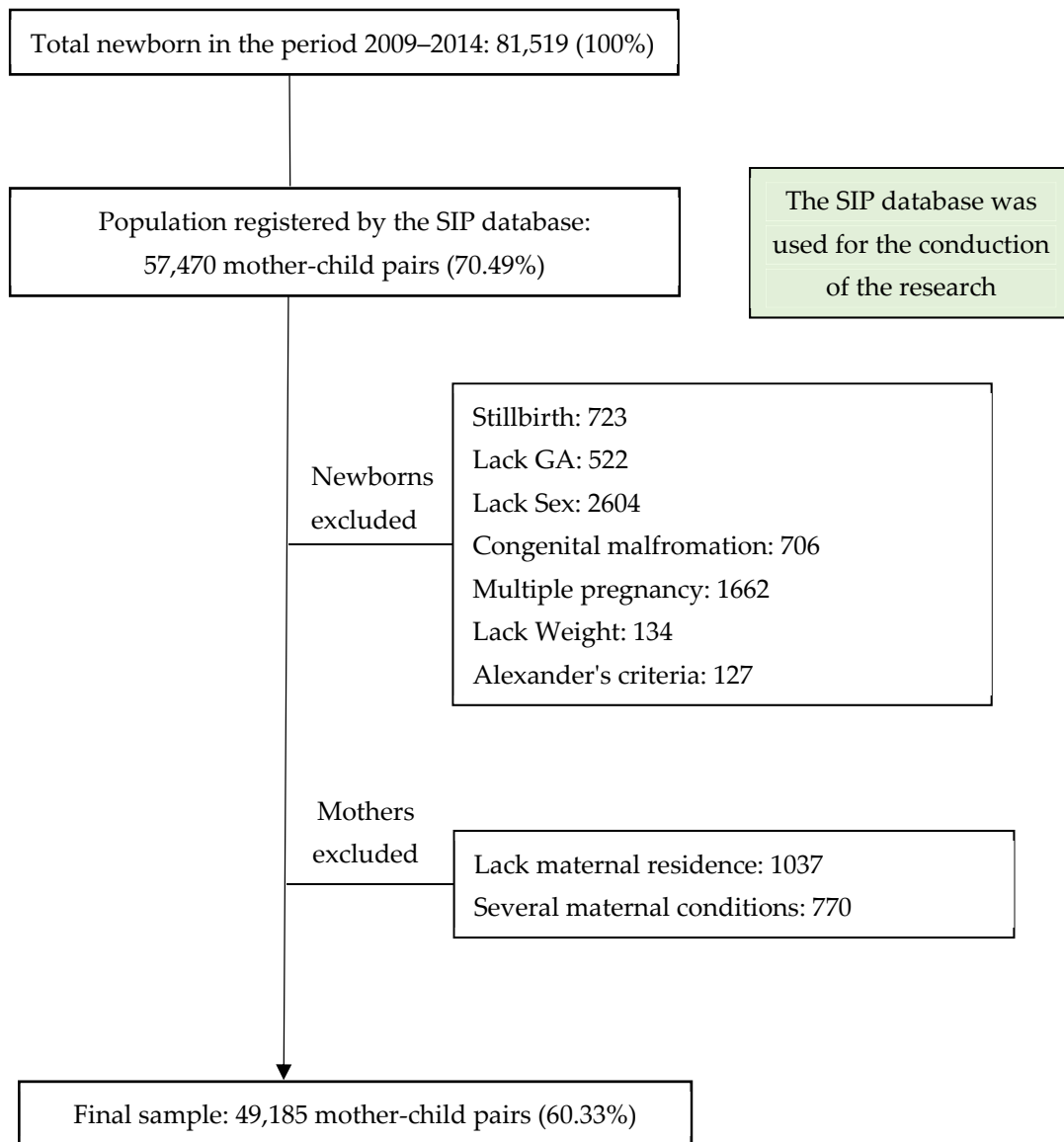

**Figure S1.** Database construction.

Supplement: Supplementary file 1 [file ijerph-19-00621-s001.zip › ijerph-1466188-supplementary.pdf]
